# Multimodal Psychophysiological Assessment of Craving in Patients With Alcohol Dependence During Virtual Reality Cue Exposure: Exploratory Single-Arm Clinical Study

**DOI:** 10.2196/84156

**Published:** 2026-06-10

**Authors:** Alva Lütt, Nadja Ruckser, Alessandro Turno, Nikolaos Tsamitros, Julia Thormann, Daniel Schulze, Ivan Nenchev, Thomas Wolbers, Sofia Marie Lange Mussons, Robert Schöneck, Jil Zoé Fuhrmann, Felix Bermpohl, Andreas Heinz, Miriam Sebold, Anne Beck, Stefan Gutwinski

**Affiliations:** 1 Department of Psychiatry and Neurosciences, Charité Campus Mitte, Charité – Universitätsmedizin Berlin, Charitépl. 1, Berlin, 10117, Germany, 49 3023112154; 2 Department of Psychiatry and Psychotherapy, Charité at St. Hedwig Hospital, Charité – Universitätsmedizin Berlin, Berlin, Germany; 3 BIH Charité Digital Clinician Scientist Program, BIH Biomedical Innovation Academy, Berlin Institute of Health at Charité – Universitätsmedizin Berlin, Berlin, Germany; 4 Partner Site Berlin-Potsdam, German Center for Mental Health (DZPG), Berlin, Germany; 5 Department of Education and Psychology, Division of Clinical Psychological Intervention, Freie Universität Berlin, Berlin, Germany; 6 Department of Neurology with Experimental Neurology, Charité – Universitätsmedizin Berlin, Campus Benjamin Franklin, Berlin, Germany; 7 Department of Psychology, Institute of Life Sciences, Humboldt-Universität zu Berlin, Berlin, Germany; 8 Institute of Biometry and Clinical Epidemiology, Charité – Universitätsmedizin Berlin, Berlin, Germany; 9 German Center for Neurodegenerative Diseases (DZNE), Magdeburg, Germany; 10 Salus Clinic Lindow, Lindow, Germany; 11 Department of Clinical Psychological Science, Faculty of Psychology and Neuroscience, Maastricht University, Maastricht, The Netherlands; 12 Department of Psychiatry and Psychotherapy, University of Tübingen, and German Center for Mental Health (DZPG) Site Tübingen, Tübingen, Germany; 13 Department of Psychiatry and Psychotherapy, Technische Universität Dresden, Dresden, Germany; 14 Department of Business and Law, TH Aschaffenburg - University of Applied Sciences, Aschaffenburg, Germany; 15 Department of Psychology, Institute for Mental Health and Behavioral Medicine, HMU Health and Medical University Potsdam, Potsdam, Germany

**Keywords:** virtual reality, alcohol dependence, craving, electrodermal activity, heart rate variability, psychophysiological parameters, pupillometry, respiration rate

## Abstract

**Background:**

Craving is a diagnostic criterion and predictor of relapse in patients with alcohol dependence (AD) and is induced in cue exposure therapy (CET) to prepare patients for real-life risk situations. The benefits of virtual reality (VR) as an innovative tool within treatment for this highly prevalent disorder include increased practicability, standardization, and personalization of CET. Accurate measurement of craving is essential to develop effective virtual reality cue exposure (VR-CE) scenarios. Despite being relevant for diagnostics and therapy, the psychophysiological reaction to alcohol cues and its relationship to subjective craving has not been sufficiently examined.

**Objective:**

This study aimed to investigate the induction of subjective craving, its physiological correlates, and their relationship in patients with AD during an innovative VR-CE paradigm, including 2 alcohol-associated risk scenarios (bar and living room) and a neutral baseline scenario.

**Methods:**

Craving was analyzed by measuring physiological reactions (electrodermal activity, including nonspecific skin conductance responses [NS-SCR] and skin conductance level [SCL]; heart rate [variability] [HR(V)]; brightness-corrected pupil diameter [BCPD]; and respiration rate [RR]) and subjective craving (visual analog scale) in 61 patients with AD. Linear mixed-effects models were conducted to estimate the effects of VR-CE. Correlations between subjective and physiological craving parameters were analyzed using Spearman correlations.

**Results:**

Results showed that alcohol-associated VR scenarios had significant effects on subjective craving (*β* values between 9.48, 95% CI 6.02‐12.95 and 15.93, 95% CI 12.47‐19.40, with moderate effect sizes between *d*=0.56 and *d*=0.72) and on NS-SCR frequency (*β* values between 0.97, 95% CI 0.14‐1.80 and 3.06, 95% CI 2.23‐3.89, with small-to-large effect sizes between *d*=0.31 and *d*=0.91), BCPD (*β* values between 0.03, 95% CI 0.01‐0.06 and 0.05, 95% CI 0.02‐0.07, with small effect sizes between *d*=0.21 and *d*=0.29), and RR (*β* values between 0.67, 95% CI 0.06‐1.28 and 1.66, 95% CI 1.05‐2.26, with small effect sizes between *d*=0.22 and *d*=0.45), but not on SCL and HR(V). Correlation analyses showed significant but weak correlations between subjective craving and electrodermal activity (SCL: *r*=0.20, *P*=.04; NS-SCR frequency: *r*=0.21, *P*=.03).

**Conclusions:**

This study showed that subjective and physiological craving (NS-SCR, BCPD, and RR but not SCL and HR[V]) can be successfully induced by VR-CE in patients with AD. These outcomes add to research on the induction of craving using a wide range of psychophysiological and subjective parameters. Furthermore, this study expands the still-limited research on the relationship between subjective and psychophysiological craving in patients with AD. In the long term, this study informs the development of effective VR-CET, which could, by further building on psychophysiological parameters, lead to biofeedback VR-CET as an innovative treatment option for patients with AD.

## Introduction

### Problem

Craving, defined as the urge to consume a psychoactive substance, is a criterion for diagnosing alcohol dependence (AD) and alcohol use disorder according to the *International Classification of Diseases, 11th Revision* (*ICD-11*) and the *Diagnostic and Statistical Manual of Mental Disorders, Fifth Edition* (*DSM-5*) [1,2] and a predictor of relapses [3]. One explanatory model, the “conditioned drug-like model,” highlights the role of substance-associated stimuli that trigger psychological and physiological responses, promoting drug-seeking behavior [4]. In cue exposure therapy (CET), a component of cognitive behavioral therapy, patients are exposed to alcohol-associated stimuli to identify risk situations and practice coping strategies, potentially reducing conditioned responses over time [5]. The effectiveness of CET for AD remains debated because of insufficient evidence, with a small number of studies and small-to-moderate effects on outcomes, underscoring the need for more trials using innovative methods such as virtual reality (VR) [6]. VR-CET has gained attention because of its advantages: realistic and immersive simulations, individualized content, increased practicability, and better standardization [7,8]. These benefits could reduce the financial and time demands of traditional in vivo CET in clinical practice [8]. Accurate craving assessment is critical for developing effective VR-CET scenarios.

### Review of Relevant Scholarship

Based on self-report data, Ghiţă et al [9] identified common triggers for alcohol craving, such as being in social settings or specific environments such as bars, restaurants, or home, and found beer, red wine, and whiskey to be most commonly associated with craving. A recent study based on this research, which implemented these VR scenarios, showed no significant differences in relapse rates between the VR-CET and control groups but did show a tendency toward lower subjective craving and anxiety in the VR-CET group. Furthermore, several important limitations of the study were highlighted, underscoring the need for further research [10]. Beyond self-reports, studies have shown that physiological responses such as pupillometry, respiration rate (RR), heart rate (HR), and electrodermal activity (EDA) can also indicate craving [11-14]. These methods help address the limitations of self-reports, including social desirability bias, defined as the tendency to give socially desirable responses [15], and recall difficulties when using retrospective reports [16]. In line with this, correlations between subjective craving and physiological responses are rather weak [17,18], but studies are few and the relationship remains ambiguous, meriting further investigation. VR may help clarify this relationship and identify physiological biomarkers relevant for diagnostics and therapy, including biofeedback applications in VR-CET in the long term.

Several studies have demonstrated that VR exposure paradigms can effectively induce craving [19]. However, research on physiological cue reactivity in VR scenarios is limited. One study included electroencephalography as a potential correlate of craving during VR-CET and showed a decrease in craving for alcohol parallel to an increase in frontal α activity over the course of the therapy [20]. One recent study by Zhang et al [12] focused on the changes in HR, EDA, and RR after VR-CET and found significantly lower increases in HR and subjective craving after cue exposure in the group receiving VR-CET. Apart from these studies, to our knowledge, further research on craving induction through virtual reality cue exposure (VR-CE) in AD populations has relied on subjective craving measurements [19].

### Hypothesis, Aims, and Objectives

In this study, we first investigated the induction and progression of craving through VR exposure using both subjective (questionnaires) and objective physiological measures (EDA, heart rate [variability] [HR(V)], brightness-corrected pupil diameter [BCPD], RR). We hypothesized that exposure to alcohol-associated VR scenarios would significantly increase (1) subjective craving and (2) physiological indicators of craving.

Additionally, we aimed to explore the relationship between subjective and objective measures, hypothesizing that (3) subjective and physiological measures of craving would show significant correlations. On an exploratory basis, we also examined the correlations between the individual psychophysiological parameters.

## Methods

### Conditions and Design

This prospective, exploratory, single-arm clinical study assessed subjective and physiological craving parameters in patients with AD. It followed a previously published study protocol with details on the study design, measures, and materials [21].

### Ethical Considerations

The study was approved by the Charité–Universitätsmedizin Berlin Institutional Review Board (EA1/190/22; May 23, 2023) and was preregistered on ClinicalTrials.gov (NCT05861843). All participants provided written informed consent and received compensation of €30 (approximately US $34.80) for participation.

All study data were pseudonymized, and identification through images or supplementary material was not possible.

### Sampling Procedures

Inpatients and outpatients diagnosed with AD were recruited at Charité–Universitätsmedizin Berlin and Salus Clinic Lindow, Germany. Patients were screened for eligibility, and they provided written informed consent and received compensation. Recruitment and data collection took place between September 23, 2023, and June 30, 2024.

### Inclusion and Exclusion

Patients aged between 18 and 65 years who were diagnosed with AD according to *ICD-10* (F10.2) based on clinical consensus were included in the study. Further inclusion criteria were completion of inpatient withdrawal treatment during the last 3 months and a history of alcohol craving.

Exclusion criteria included other substance dependencies (except nicotine), less than 7 days of abstinence or current intoxication (randomly tested using breath alcohol concentration), severe neuropsychiatric disorders (eg, schizophrenia and bipolar disorder), cognitive impairment, acute suicidality or risk of endangering others, serious conditions affecting brain or heart function, current use of AD-targeting medication (eg, benzodiazepines and acamprosate), medications significantly affecting HR, or inability to understand study procedures.

### Data Collection

#### Clinical Data and Questionnaires

Clinical data and questionnaires were collected using REDCap (Research Electronic Data Capture) tools hosted at Charité–Universitätsmedizin Berlin [22,23].

General baseline data included demographic information, medical history, comorbidities (assessed using Charlson Comorbidity Index [24] and patient information), and medication. Subjective craving was measured using a 0 to 100 visual analog scale (VAS) [25] at 3 time points (0:30, 2:30, and 4:30 min) in each VR scenario using verbal reports from participants [21]. The study workflow followed the previously published protocol [21].

#### VR Exposure

VR exposure was conducted according to the study protocol, for which a detailed description can be found in Multimedia Appendix 1 [21]. The hardware consisted of a VR head-mounted display (HTC VIVE Pro Eye) and a desktop PC based on the SCHENKER XR Station with Intel Core i5-12,500. Patients selected a preferred drink (schnapps, red wine, white wine, beer, or vodka) and a bar environment (wine bar or corner pub), and scenarios were personalized accordingly. In addition to the bar environment, a second risk scenario featured a living room setting with the drink of choice. All patients started with a VR acclimation session (1‐5 min) consisting of a white waiting room for acclimatization to VR before proceeding to a neutral baseline scenario (5 min). This room was a black space with a grid for spatial orientation. Next, patients entered the first of 2 risk scenarios presented in randomized order (5 min each). After a 45-minute break, the second baseline-risk paradigm was conducted. After completing the final questionnaires following VR exposure, patients were asked to confirm that they could safely leave the study site without experiencing an increased risk of relapse. Immediate therapeutic support would have been provided if necessary, but it was not required in any case. The VR sessions were conducted by 2 medical doctors and 1 psychologist with experience in VR applications and treatment of psychiatric patients.

#### Psychophysiological Data

Psychophysiological data were collected using the Bionomadix wireless respiration and electrocardiography (ECG), photoplethysmography, and EDA amplifiers in combination with the Biopac MP160 data acquisition system (Biopac Systems). The system was connected to an acquisition computer running the AcqKnowledge 5.0 software (Biopac Systems). Signals were obtained using a 1000 Hz sampling rate.

#### Electrodermal Activity

Disposable Ag/AgCl dry electrodes were prepared with isotonic paste and placed on the volar surfaces of the medial phalanges of the index and middle fingers of the nondominant hand. EDA was measured by assessing the slow-varying skin conductance level (SCL) and the frequency of nonspecific skin conductance responses (NS-SCR) exceeding a threshold of 0.03 μS. We excluded participants who failed to show a peak above the threshold of 0.03 from the analysis of NS-SCR frequency (“nonresponders” according to established guidelines) [26,27]. The SCL and NS-SCR frequency were extracted from the EDA signal using continuous decomposition analysis via the Ledalab toolbox [28] for MATLAB (MathWorks), version 24.2.0.2712019 [29].

#### Heart Rate and Heart Rate Variability

HR and heart rate variability (HRV) were obtained via a 3-lead ECG. Electrodes were placed on the chest 1 cm below the sternum, left and right, along the midclavicular line, and 1 cm left of the parasternal line, between the fifth and sixth left sternochondral joints. Before performing the analysis, each waveform was preprocessed within the AcqKnowledge HRV Suite, using the Biopac’s HR detection algorithm. Applying Biopac’s Classify Script—an automated ECG beat-detection and derivative-based QRS peak realignment algorithm—to the waveform within the AcqKnowledge environment, intervals between successive heartbeats (R-R intervals) were computed and labeled, flagging out-of-range values or significant deviations from neighboring values. Waveforms were visually inspected for artifacts and manually corrected by 2 medical experts to allow differentiation between artifacts and actual arrhythmias. Artifacts were replaced with an aligned R marker, calculating the midpoint between 2 existing QRS markers and inserting a new marker at the estimated equidistant position in the ECG signal. Using the preprocessed signal, various time-domain, frequency-domain, and nonlinear-domain parameters were extracted using the AcqKnowledge HRV Analysis Suite for the whole 5-minute duration of each VR scenario.

The time-domain parameters included the standard deviation (SD) of successive differences in R-R intervals (SDSD, ms), the root mean square of successive R-R differences (RMSSD, ms), and the percentage of successive R-R intervals differing by more than 50 ms (pNN50, %). The frequency domain included the power as measured in the following bands (ms^2^): the very low frequency band (VLFB, 0.0033‐0.04 Hz), the low frequency (LF) band (LFB, 0.04‐0.15 Hz), the high frequency (HF) band (HFB, 0.15‐0.40 Hz), and the very high frequency band (VHFB, 0.40‐3.00 Hz). Additionally, total power (ms^2^), LF/(LF+HF), HF/(LF+HF), and LF/HF ratios were calculated. For the nonlinear domain, the SD of each point from the *y*=x axis (SD1), the SD of each point from the *y*=*x*+average R-R interval (SD2), and the SD1/SD2 ratio were calculated. The cleaned R-R intervals were processed using Python 3.9.20 with the pandas, NumPy, and regex packages [30-32], extracting the SD of R-R intervals (SDRR, ms, time-domain parameter) and obtaining HR by averaging the R-R intervals within each 1-minute segment of the exposure period.

#### Brightness-Corrected Pupil Diameter

Pupil diameter and scene brightness in VR were recorded continuously during the paradigm using the built-in eye-tracking device within the VIVE Pro Eye Headset (120 Hz binocular data output frequency, 110° trackable field of view). Data were processed using the Soma Reality algorithm (Soma Reality GmbH), which differentiates between illumination-induced and cognitive-induced (“cognitive load”) components of pupil diameter variation, as shown by Gollan [33] and based on Beatty et al [34]. The approach combines (1) a brightness measure of the content displayed in the VR headset, (2) an empirical model of the pupillary light reflex, and (3) a temporal modeling of the delay of the pupillary response to model how the pupil size reacts to perceived brightness. This allows for the separation of light-induced effects from cognitive effects in pupillary behavior and the extraction of BCPD. Preprocessing was conducted in Jupyter Notebook, version 7.0.8 [35], using the Anaconda Python distribution, version 24.11.3 [36], and the pandas and NumPy packages [30-32]. Confidence scores for BCPD values were calculated.

#### Respiration Rate

The respiratory transducer was attached to the chest or abdomen using a fully adjustable nylon strap, depending on the primary breathing pattern. The respiratory signal was filtered using a bandpass finite impulse response filter between 0.05 and 1 Hz. Breaths per minute were then extracted for further analysis of RR.

### Analytic Strategy

All analyses were performed using R software (version 4.3.2, Eye Holes; R Foundation for Statistical Computing) [37] and RStudio (version 2024.4.0.735, Chocolate Cosmo; Posit) [38]. Demographic and baseline characteristics are reported as mean, SDs, and ranges.

EDA, HR, BCPD, and RR data were divided into 5 equal segments for each 5-minute exposure period (baseline: B1, B2, B3, B4, and B5; risk scenario: R1, R2, R3, R4, and R5). HRV parameters were calculated across each entire VR scenario (5 min; baseline: B, risk scenario: R). Each exposure combined a baseline and a risk scenario into 1 data frame. Because each participant underwent 2 exposures, each participant produced 2 data frames (N=61 participants, N=122 data frames). Because of recording problems, some data were unavailable: one participant lacked all physiological recordings, resulting in 2 missing data frames for BCPD, RR, EDA parameters, HR, and HRV. In addition, 4 BCPD data frames from 3 participants were missing, leaving 116 data frames for BCPD analysis. For HRV, 1 additional data frame with partial missing values (segments R4 and R5) was excluded, resulting in 119 data frames for HRV analysis. NS-SCR frequency was calculated from 116 data frames, with 2 participants excluded as nonresponders.

Subjective craving, measured using VAS, was assessed during first (B1 and R1), third (B3 and R3), and fifth (B5 and R5) segments, with one missing data frame, resulting in 121 data frames.

The main analyses were conducted using linear mixed-effects models fitted with the nlme package (version 0.3.2) [39] to estimate the effects of VR-CE. Models were specified with a categorical variable for craving assessment as a fixed effect, indicating exposure to baseline (B1-B5) and risk scenarios (R1-R5). A random intercept was included to account for individual variability, whereas a fixed slope was used to estimate the overall effect across all participants. The mean baseline value (B) was included as the reference category. Accordingly, the estimated fixed effects (*β*) from the linear mixed-effects models are reported as model-based mean differences between each craving assessment and the baseline assessment, as estimated by the nlme package. Models are reported in adjusted form, using covariates with influence on the psychophysiological parameters identified a priori. These covariates were selected individually based on guidelines and prior research and were centered within cluster. Covariates for EDA included gender, age, ambient temperature, and medication [27]. In addition to these covariates, BMI, smoking status, and health status (Charlson Comorbidity Index score) were incorporated for HR, HRV [40], and RR [41-45]. Medication and age were also included for BCPD [46-51]. Ambient temperature for 1 participant was interpolated because of a missing value. Post hoc calculations of *t* statistics and *P* values were performed using the Satterthwaite approximation [52]. Missing data were handled using linear mixed-effects models, which incorporate all available data from each participant into the analysis. Effect sizes were estimated as standardized mean differences, that is, approximations of Cohen *d* based on mixed-effects modeling following Pustejovsky et al [53], using the *lmeInfo* (version 0.3.2) [54] and *nlme* (version 0.3.2) [39] packages. Plots of the estimated model effects were generated using the *effects* package (version 4.2.4) [55]. Correlations between subjective and physiological craving parameters were analyzed using Spearman correlations on calculated difference scores of the parameters between baseline and risk scenarios (first segment: R1-B1; second segment: R2-B2; third segment: R3-B3; fourth segment: R4-B4; and fifth segment: R5-B5). Correlation analyses could only be conducted using complete data frames, leading to 111 data frames for analysis. To correlate subjective and physiological parameters at the same time points, physiological measures during the first, third, and fifth segments of the exposure were selected, during which subjective craving was assessed. Additional correlation analysis was conducted for psychophysiological craving parameters (EDA, HR, RR, and BCPD), excluding HRV because of the longer time frames for analyses. Effect sizes are reported as Cohen *d*, with 0.2 considered as small, 0.5 as moderate, and 0.8 as large [56]. The predetermined *α* level was .05. This study was reported in accordance with the American Psychological Association Reporting Standards for quantitative research [57].

## Results

### Descriptive Analysis

Baseline demographic and medical characteristics are presented in Table 1. Participants (49/61, 80% male and 12/61, 20% female) had a mean age of 48.16 (SD 9.82; range 31‐65) years, with a mean duration of 20.64 (SD 22.42; range 7‐109) days since their last alcohol consumption on the day of the experiment.

**Table 1.** Baseline demographic and medical characteristics.

Variables	Values
Age (years), mean (SD; range)	48.16 (9.82; 31‐65)
Sex, n (%)	
Male	49 (80)
Female	12 (20)
BMI, mean (SD; range)	26.92 (5.84; 18.8‐48.8)
Medical baseline data, mean (SD; range)	
Days since last alcohol consumption	20.64 (22.42; 7‐109)
Alcohol consumption in the past 5 years (kg)	342.70 (301.64; 112.26‐1421.12)
Phases with moderate drinking	251.35 (211.34; 8.19‐835.52)
Phases with maximum drinking	91.34 (153.39; 0‐756.10)
Comorbidities	
Yes, n (%)	38 (62)
CCI^a^ score, age adjusted, mean (SD; range)	1.59 (1.37; 0‐5)
CCI score, mean (SD; range)	0.93 (1.08; 0‐4)
No, n (%)	23 (38)
Medication, n (%)	
Yes	39 (64)
No	22 (36)
Detoxification	
Once, n (%)	17 (28)
More than once, n (%)	44 (72)
Number of completed detoxifications, mean (SD; range)	5.72 (6.86; 0‐25)
Number of discontinued detoxifications, mean (SD; range)	0.89 (2.47; 0‐15)
Number of completed addiction (long-term) rehabilitation therapies, mean (SD; range)	0.95 (1.19; 0‐5)
Number of discontinued addiction (long-term) rehabilitation therapies, mean (SD; range)	0.23 (0.67; 0‐4)
Consumption of drugs besides alcohol and tobacco, n (%)	
Yes	29 (48)
No	32 (52)
Smoking	
Yes, n (%)	45 (74)
Pack years, mean (SD; range)	29.06 (16.16; 5‐94)
Attempts quitting, n (%)	
Never	14 (31)
Once	13 (29)
More than once	18 (40)
No, n (%)	16 (26)

a CCI: Charlson Comorbidity Index.

**Figure 1.**
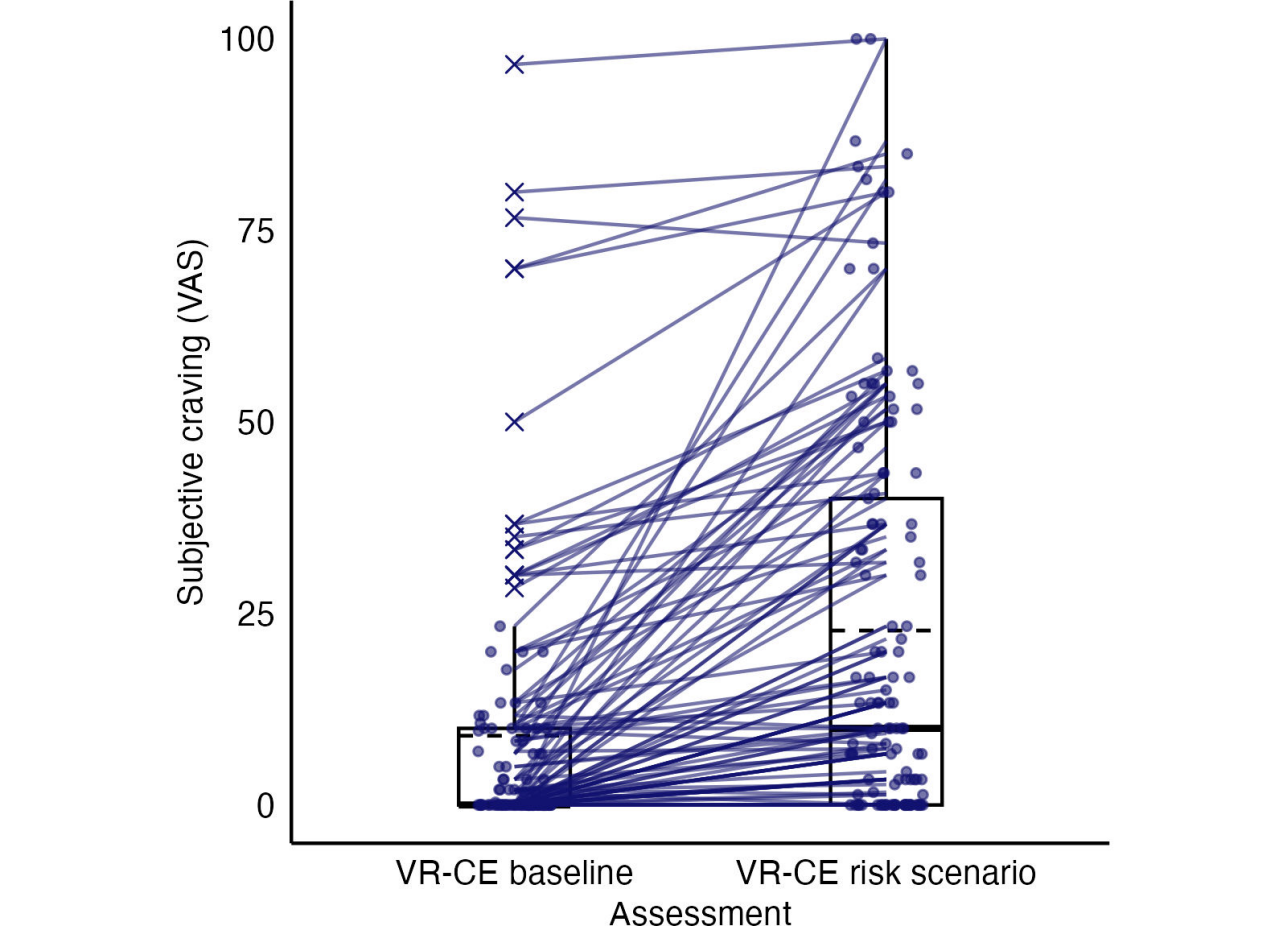
Subjective craving measured using the visual analog scale (VAS) during virtual reality cue exposure (VR-CE).

Mean craving was 9.03 (SD 17.96) during the VR-CE baseline and 22.77 (SD 26.49) during the risk scenario, as assessed using a VAS ranging from 0 to 100 (Figure 1). Cybersickness, including any sign of discomfort, nausea, and dizziness, during the VR-CE was reported by 67.2% (41/61) of patients on the Fast Motion Sickness Scale.

Figure 1 shows subjective craving measured by VAS during VR-CE baseline and risk scenarios, displayed using a combination of boxplots and spaghetti plots. The boxplots display the mean (dotted line), median (horizontal line), IQR (box), whiskers (1.5 × IQR), and outliers marked as crosses.

### Statistics and Data Analysis

#### Effects of VR-CE on Subjective and Physiological Parameters

The linear mixed-effects model analysis showed an overall significant main effect of the VR-CE on subjective craving as indicated by the VAS, indicating significantly higher craving observed in the risk scenarios, with a mean difference to baseline of 9.48 (95% CI 6.02‐12.95; R1) during the first segment, 15.93 (95% CI 12.47‐19.40; R3) during the third segment, and 15.82 (95% CI 12.35‐19.28; R5) during the fifth segment of the risk scenarios. This corresponds to moderate effect sizes between *d*=0.56 (R1) and *d=*0.72 (R3) for the VR-CE. Further results are displayed in Table S1 in Multimedia Appendix 1.

**Figure 2.**
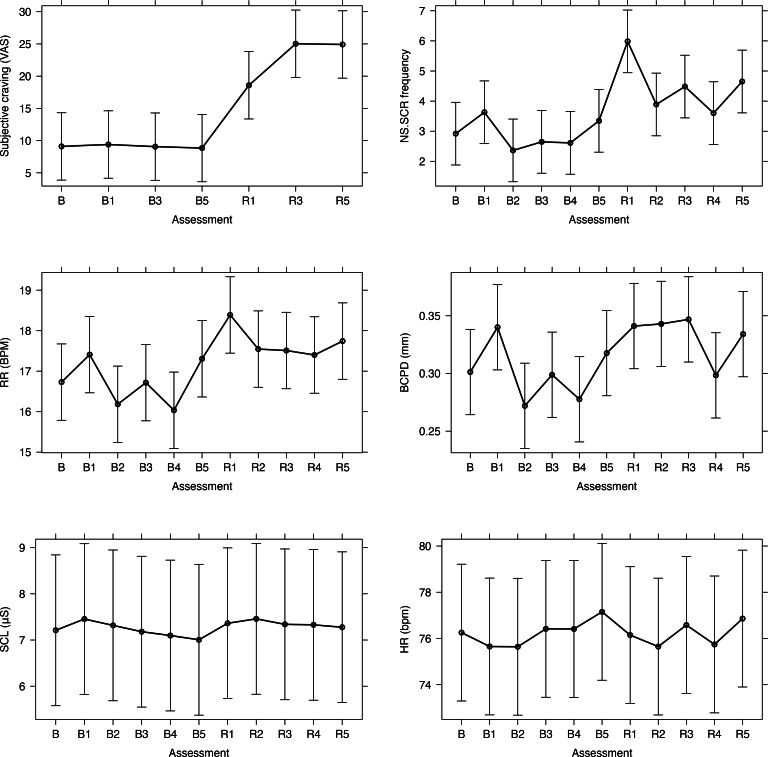
Effects of virtual reality cue exposure on subjective and physiological parameters. BCPD: brightness-corrected pupil diameter; bpm: beats per minute; BPM: breaths per minute; HR: heart rate; NS-SCR: nonspecific skin conductance responses; RR: respiration rate; SCL: skin conductance level; VAS: visual analog scale.

For the physiological craving parameters, the analyses showed an overall significant effect of the VR-CE on NS-SCR frequency, with a mean difference to baseline between 0.97 (95% CI 0.14‐1.80; R2) and 3.06 (95% CI 2.23‐3.89; R1), with small-to-large effect sizes between *d*=0.31 (R2) and *d*=0.91 (R1). However, the fourth segment of the risk scenarios did not show statistical significance (0.68, 95% CI −0.15 to 1.51; R4). Furthermore, analyses showed an overall significant effect of the VR-CE on RR, with a mean difference to baseline between 0.67 (95% CI 0.06‐1.28; R3) and 1.66 (95% CI 1.05‐2.26; R1), indicating small effect sizes between *d*=0.22 (R3) and *d*=0.45 (R1). We also observed an overall significant effect of the VR-CE on BCPD, with a mean difference to baseline between 0.03 (95% CI 0.01‐0.06; R5) and 0.05 (95% CI 0.02‐0.07; R3) and small effect sizes between *d*=0.21 (R5) and *d*=0.29 (R1), except for the fourth segment of the risk scenarios (−0.00, 95% CI −0.03 to 0.02; R4). The result for the main effect of the VR-CE on BCPD during the risk scenarios remained consistent after excluding datasets of BCPD in which confidence levels fell below 0.3 for phases exceeding 30 seconds or in which more than 20% were below this threshold. No significant effect of the VR-CE on SCL, HR, or any of the HRV parameters was detected (for details see Figure 2; Tables S1-S3 and Figure S1 in Multimedia Appendix 1). Figure 2 shows plots of estimated marginal means and 95% model-based CIs displaying main effects of the VR-CE over time on subjective craving and physiological craving parameters. The figure depicts the segments for baseline (B1-B5) and risk scenario (R1-R5), with the mean of baseline as the reference category for this analysis.

#### Correlation of Subjective and Physiological Craving Parameters

**Figure 3.**
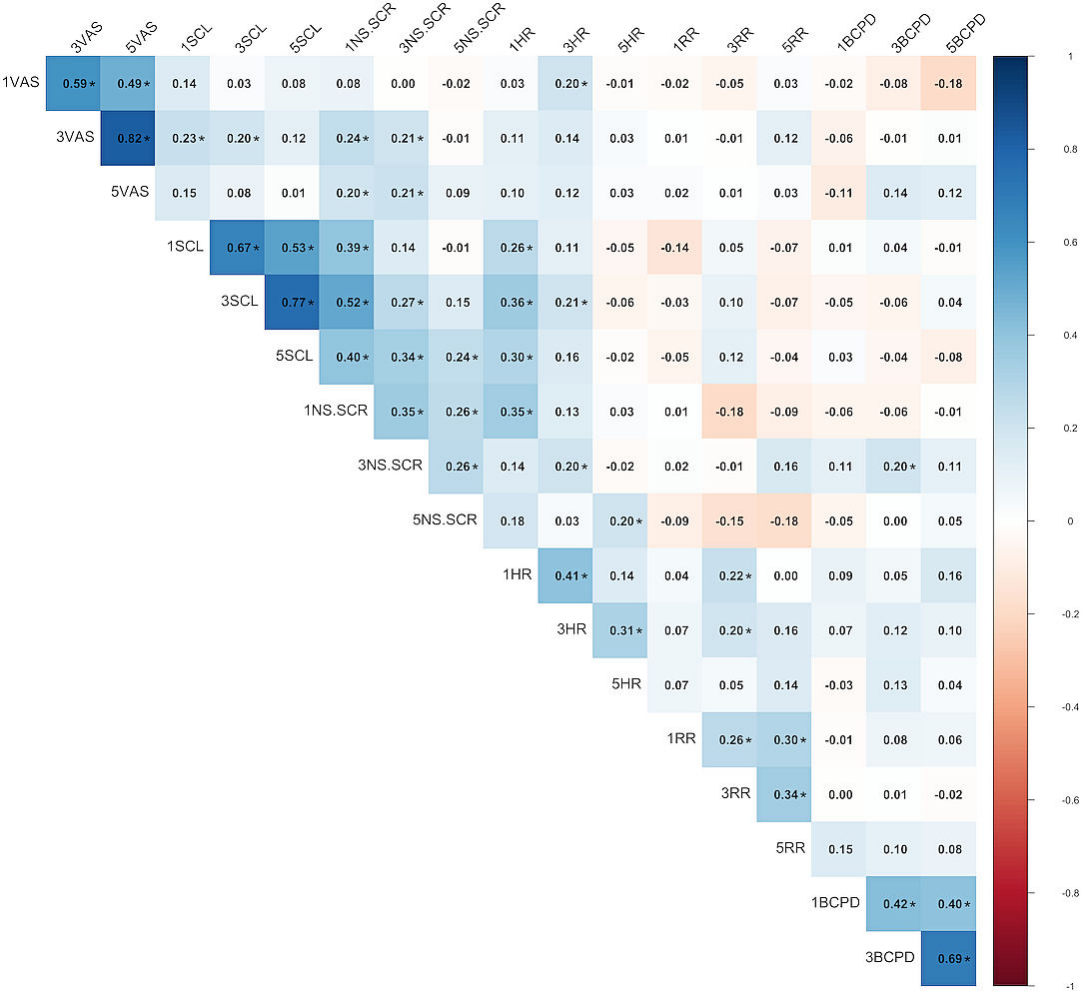
Correlation heatmap of subjective and physiological craving parameters. BCPD: brightness-corrected pupil diameter; HR: heart rate; NS-SCR: nonspecific skin conductance responses; RR: respiration rate; SCL: skin conductance level; VAS: visual analog scale.

The correlation analysis revealed no significant relationships between subjective and physiological craving parameters during simultaneous segments of the assessments (*P*>.05; Figure 3 and Table S3 in Multimedia Appendix 1), except for the third segment of the assessment of subjective craving and the EDA parameters SCL (*r*=0.20, *P=*.04; see Table S4 in Multimedia Appendix 1) and NS-SCR frequency (*r*=0.21, *P*=.03). Significant correlations with small effect sizes were also observed between nonsimultaneous assessments of subjective craving and NS-SCR frequency.

Significant correlations of time-corresponding segments of the assessment between the physiological craving parameters were identified *(P<.*05; see Figure 3 and Table S3 in Multimedia Appendix 1). The parameters of EDA, SCL and NS-SCR frequency showed significant correlations for all segments of the assessment between *r*=0.24 (*P*=.01; fifth segment) and *r*=0.39 (*P*<.001; first segment). EDA parameters further showed significant correlations with HR: in segments 1 to 3 of the assessment of SCL and HR, ranging between *r*=0.21 (*P*=.03; third segment) and *r*=0.29 (*P*=.002; second segment), and in the first segment as well as segments 3 to 5 of the assessment of NS-SCR frequency and HR, ranging between *r*=0.20 (*P*=.03, third segment and *P*=.04, fifth segment) and *r*=0.35 (*P*<.001; first segment). NS-SCR frequency further showed a correlation with RR during the second segment of the assessment (*r*=−0.19*, P*=.04) and with BCPD during the third segment of the assessment (*r*=0.20, *P*=.04), whereas SCL showed no significant correlations with RR and BCPD (*P*>.05). Furthermore, HR and RR showed significant correlations during the third (*r*=0.20, *P*=.04) and fourth (*r*=0.21, *P*=.03) segments of the assessment. There were no significant correlations with BCPD and HR as well as RR (*P*>.05). Significant correlations of time-corresponding segments of the assessment all corresponded to small effect sizes. Figure 3 shows a correlation heatmap of subjective craving measured and physiological craving parameters (NS-SCR, RR, and BCPD). Significant correlations are labeled with an asterisk. HRV parameters are not depicted because of the longer analysis time frames.

## Discussion

### Principal Findings

This study demonstrates that exposure to alcohol-associated VR scenarios significantly induced subjective craving. VR-CE had significant effects on NS-SCR as one measure of EDA, on BCPD, and on RR. No significant changes were observed in SCL, as a second EDA parameter, HR, or HRV. Our first hypothesis was thus confirmed and our second hypothesis partially confirmed. Correlation analyses focusing on simultaneous time segments revealed significant but weak correlations between subjective craving and EDA measures. Subjective craving was not significantly correlated with other physiological parameters. Additionally, significant correlations were found in simultaneous assessments between several physiological craving parameters, leading to a partial confirmation of our third hypothesis.

### Subjective Craving

This study confirms that VR effectively induces subjective craving, consistent with positive results from previous studies [9,58,59]. A recent scoping review from our group once again confirmed that the majority of studies report significant changes in craving [60]. The current study builds on a prior feasibility study [61], adding comparisons to baseline scenarios and physiological measures. Observed effect sizes (*d*=0.56‐0.72) indicate moderate effects, higher than those reported in the meta-analysis by Carter and Tiffany [62] (*d*=0.53). This may be due to the higher ecological validity of VR compared with more conventional stimuli (eg, images, sounds, or in vivo cues) used in prior studies [62].

### Psychophysiological Craving

The results of our study indicate that measures of EDA (NS-SCR), BCPD, and respiration could serve as sensitive biomarkers of craving in patients with AD exposed to high-risk scenarios in VR. These measures provide objective insight into a subjective, bias-prone construct and may support more effective VR-CET, including biofeedback integration. Providing patients with feedback on their physiological data may help them better recognize and manage personal risk cues [63].

To date, we are aware of only 2 other VR craving studies that include psychophysiological measures of craving in patients with AD. However, their designs differed from ours: Lee et al [20] reported an increase in electroencephalography α power and a greater craving reduction through 10 sessions of VR-CET compared with patients receiving cognitive therapy. Zhang et al [12] compared 2 groups (n=23 vs n=21) of male patients with AD, one group receiving treatment as usual and the other group receiving additional VR-CET sessions. Comparing these 2 groups regarding their cue reactivity in VR assessments, the VR group exhibited significantly lower changes in subjective craving and HR, but not in EDA and RR, after treatment [12]. Notably, in the study by Zhang et al [12], skin conductance was averaged across the whole signal and not evaluated separately (eg, SCL, SCR, or NS-SCR), which could artificially elevate the parameter [12,26,27]. Although we employed a different analysis approach, we also found no significant effects of VR-CE on SCL. In contrast to Zhang et al [12], we did not observe significant changes in HR but did note changes in RR. Additionally, we used HRV and BCPD, expanding the physiological scope. While VR-CE had significant effects on BCPD, this was not the case for HRV parameters. Previous studies have reported inconsistent conclusions on this matter: Wang et al [64] observed higher RMSSD in patients with AD during exposure to alcohol cues compared with neutral cues. High-frequency HRV, a marker of parasympathetic activity, has been shown to react to alcohol-associated cues and is associated with relapse in patients with AD [65,66]. It may be worth considering that VR itself may elevate HR and SCL because of sensory stimulation, potentially obscuring craving-specific responses.

### Relation Between Subjective and Physiological Craving

Similar to our study, several studies indicated that subjective craving is only weakly associated with physiological parameters. For instance, a meta-analysis conducted by Field et al [18] on craving in various substance-use disorders found low correlations (*r*=0.19) between subjective craving and attentional bias. Similarly, Witteman et al [67] reported low-to-moderate correlations between HRV and subjective craving (*r*=0.33, *P=*.004). Interestingly, Szegedi et al [68] identified 3 groups of patients with AD: one with both subjective and physiological craving responses, another group with only physiological craving responses, and a third group with no response at all. In contrast, Ooteman et al [17] found only a few patients with or without physiological craving responses who also reported subjective craving, suggesting a need to explore possibilities to help patients become more aware of these sensations. Analyses by Bollen et al [69,70] and Field et al [71] showed that patients with AD who report only low subjective craving and high abstinence motivation tend to avoid alcohol-associated cues when presented. Based on these findings, future studies should include fixation analyses as a measure of attentional bias to better understand the relationship between subjective craving and physiological parameters and why these sometimes differ from one another.

### Limitations

The single-arm design of this study does not allow comparisons between patients with AD and healthy controls, which would have shifted the study’s focus but should be reconsidered in further studies. Regarding the physiological measurements, we did not assess nicotine or caffeine consumption directly before study participation, which could have influenced both the baseline and risk scenario results. Additionally, while gender and age were shown to influence physiological parameters such as EDA, our sample’s gender distribution reflects AD prevalence and was adjusted for in the models. Further studies should include additional nonalcohol cue exposure scenarios that are comparable in complexity to the alcohol cue exposure scenarios because the complexity might, for example, influence BCPD. This will strengthen confidence in the ability of specific physiological parameters to indicate alcohol craving. A further control group exposed to alcohol-associated stimuli outside VR (eg, photographs) could be used to correct for the potential sensory stimulation of VR described above.

### Conclusions

We demonstrated that subjective craving can be successfully induced by VR-CE in patients diagnosed with AD. NS-SCR, RR, and BCPD were significantly altered, reflecting a physiological craving response, whereas HR, HRV, and SCL did not react significantly to alcohol-associated cues. Correlation analyses indicated significant but weak correlations between subjective craving and EDA. Based on high methodological standards and a wide range of psychophysiological craving parameters, this study complements the ambiguous research situation regarding the induction of subjective and physiological craving and the relationship between subjective and psychophysiological parameters, offering guidance for future research on craving in VR-CE. In the long term, this will inform the development of effective VR-CET, and through further focus on psychophysiological craving signatures, possibly also biofeedback-based VR-CET in patients with AD, a disorder for which innovative treatment options are urgently needed.

## Supplementary material

10.2196/84156 Multimedia Appendix 1 Results of linear mixed model analysis of main effects and correlation matrix table.

## Abbreviations

AD: alcohol dependence
BCPD: brightness-corrected pupil diameter
CET: cue exposure therapy
*DSM-5*: *Diagnostic and Statistical Manual of Mental Disorders, Fifth Edition*
ECG: electrocardiography
EDA: electrodermal activity
HF: high frequency
HFB: high frequency band
HR: heart rate
HRV: heart rate variability
*ICD-11*: *International Classification of Diseases, 11th Revision*
LF: low frequency
LFB: low frequency band
NS-SCR: nonspecific skin conductance responses
pNN50: percentage of successive R-R intervals differing by more than 50 ms
REDCap: Research Electronic Data Capture
RMSSD: root mean square of successive R-R differences
RR: respiration rate
SCL: skin conductance level
SDRR: standard deviation of R-R intervals
SDSD: standard deviation of successive differences in R-R intervals
VAS: visual analog scale
VHFB: very high frequency band
VLFB: very low frequency band
VR: virtual reality
VR-CE: virtual reality cue exposure
