# Supplementary material for: Multimodal Psychophysiological Assessment of Craving in Patients With Alcohol Dependence During Virtual Reality Cue Exposure: Exploratory Single-Arm Clinical Study
Source: JMIR Serious Games. 2026 Jun 10;14:e84156. doi: 10.2196/84156 (PMC13252710; doi:10.2196/84156)
Supplement: Multimedia Appendix 1 [file games-v14-e84156-s001.pdf]

Supplementary Material

Supplementary Table 1. Results of linear mixed model analysis of main effect of VR-CE on subjective craving parameters and SCL, NS.SCR frequency, HR, RR and BCPD.

		**VAS**					**SCL**[1]				**NS.SCR frequency**[1]			
			Linear mixed effects model analysis					Linear mixed effects model analysis				Linear mixed effects model analysis		
		Mean (SD)	β (95% CI)	*p*–value	Effect size[4]		Mean (SD)		*p*–value	Effect size[4]	Mean (SD)	β (95% CI)	*p*–value	Effect size[4]
Assessment[5]	B	9.03 (17.96)				B	7.21 (6.57)				2.92 (3.76)			
	B1	9.32 (17.86)	0.29 (−3.17, 3.76)	.867	0.07	B1	7.45 (6.65)	0.24 (−0.07, 0.55)	.123	0.74	3.63 (4.75)	0.71 (−0.12, 1.54)	.094	0.38
						B2	7.32 (6.63)	0.11 (−0.20, 0.42)	.502	0.47	2.36 (3.79)	−0.56 (−1.39, 0.27)	.189	−0.44
	B3	8.99 (18.32)	−0.04 (−3.50, 3.43)	.984	−0.02	B3	7.18 (6.58)	−0.03 (−0.34, 0.28)	.847	−0.23	2.65 (2.98)	−0.27 (−1.10, 0.56)	.520	−0.14
						B4	7.10 (6.54)	−0.11 (−0.42, 0.20)	.472	−0.60	2.61 (5.24)	−0.31 (−1.14, 0.52)	.469	−0.14
	B5	8.77 (18.56)	−0.26 (−3.72, 3.21)	.883	−0.08	B5	7.00 (6.50)	−0.21 (−0.51, 0.10)	.193	−0.53	3.34 (3.77)	0.43 (−0.40, 1.26)	.315	0.25
	R1	18.51 (25.19)	9.48 (6.02, 12.95)	<.001	0.56	R1	7.36 (6.73)	0.15 (−0.16, 0.46)	.336	0.26	5.98 (5.98)	3.06 (2.23, 3.89)	<.001	0.91
						R2	7.46 (6.75)	0.25 (−0.06, 0.56)	.117	0.26	3.89 (4.60)	0.97 (0.14, 1.80)	.022	0.31
	R3	24.96 (28.63)	15.93 (12.47, 19.40)	<.001	0.72	R3	7.34 (6.67)	0.13 (−0.18, 0.44)	.415	0.14	4.48 (5.94)	1.56 (0.73, 2.39)	<.001	0.33
						R4	7.36 (6.68)	0.12 (−0.19, 0.43)	.455	0.14	3.63 (5.25)	0.68 (−0.15, 1.51)	.109	0.20
	R5	24.84 (30.00)	15.82 (12.35, 19.28)	<.001	0.67	R5	7.31 (6.65)	0.07 (−0.24, 0.38)	.678	0.14	4.68 (6.07)	1.73 (090, 2.56)	<.001	0.33

	**HR**[2]				**RR**[2]				**BCPD**[3]			
		Linear mixed effects model analysis				Linear mixed effects model analysis				Linear mixed effects model analysis		
	Mean (SD)	β (95% CI)	*p*–value	Effect size[4]	Mean (SD)	β (95% CI)	*p*–value	Effect size[4]	Mean (SD)	β (95% CI)	*p*–value	Effect size[4]
B	76.21 (12.38)				16.73 (3.72)				0.30 (0.16)			
B1	75.61 (12.38)	−0.60 (−1.87, 0.67)	.353	−0.34	17.41 (4.23)	0.68 (0.07, 1.28)	.028	0.39	0.34 (0.17)	0.04 (0.01, 0.07)	.006	0.56
B2	75.60 (12.59)	−0.61 (−1.89, 0.66)	.344	−0.44	16.18 (4.45)	−0.55 (−1.15, 0.06)	.076	−0.30	0.27 (0.17)	−0.03 (−0.06, −0.00)	.039	−0.45
B3	76.37 (12.42)	0.16 (−1.11, 1.43)	.803	0.17	16.71 (3.89)	−0.01 (−0.62, 0.59)	.967	0.00	0.30 (0.18)	−0.00 (−0.03, 0.03)	.869	−0.04
B4	76.37 (12.53)	0.16 (−1.12, 1.43)	.810	0.13	16.03 (4.04)	−0.70 (−1.30, −0.09)	.025	−0.34	0.28 (0.18)	−0.02 (−0.05, 0.00)	.097	−0.38
B5	77.11 (12.39)	0.90 (−0.37, 2.17)	.166	0.54	17.30 (4.06)	0.58 (−0.03 1.18)	.062	0.30	0.32 (0.17)	0.02 (−0.01, 0.04)	.248	0.24
R1	76.10 (11.79)	−0.11 (−1.38, 1.16)	.867	−0.02	18.38 (3.94)	1.66 (1.05, 2.26)	<.001	0.45	0.34 (0.15)	0.04 (0.01, 0.07)	.005	0.29
R2	75.61 (11.80)	−0.61 (−1.88, 0.67)	.351	−0.09	17.54 (4.15)	0.82 (0.21, 1.42)	.008	0.28	0.34 (0.16)	0.04 (0.01, 0.07)	.003	0.25
R3	76.54 (11.56)	0.33 (−0.94, 1.60)	.614	0.05	17.51 (4.00)	0.78 (0.17, 1.39)	.012	0.24	0.35 (0.16)	0.05 (0.02, 0.07)	.001	0.26
R4	75.70 (11.83)	−0.51 (−1.79, 0.76)	.430	−0.07	17.37 (4.11)	0.67 (0.06, 1.28)	.031	0.22	0.30 (0.14)	−0.00 (−0.03, 0.02)	.840	−0.02
R5	76.82 (11.55)	0.61 (−0.67, 1.88)	.351	0.11	17.72 (4.27)	1.01 (0.41, 1.62)	.001	0.29	0.34 (0.15)	0.03 (0.01, 0.06)	.021	0.21

[1] Gender, age, ambient temperature and medication included as covariates in the linear mixed model

[2] Gender, age, ambient temperature, medication, cci-score and smoking included as covariates in the linear mixed model

[3] Age and medication included as covariates in the linear mixed model

[4] Cohens d

[5] Segments for baseline (B1–B5) and risk scenario (R1–R5) with the mean of baseline (B) as the reference category for this analysis

VAS: visual analogue scale, B: baseline, R: risk scenario, SCL: skin conductance level, NS.SCR: non-specific skin conductance responses, HR: heart rate, RR: respiration rate, BCPD: brightness corrected pupil diameter

Supplementary Table 2. Results of linear mixed model analysis of main effect of VR-CE on BCPD without the BCPD data sets in which confidence levels fell below 0.3 for phases exceeding 30 seconds or in which more than 20% were below this threshold.

		**BCPD**[1]			
			Linear mixed effects model analysis		
		Mean (SD)	β (95% CI)	*p*–value	Effect size[2]
Assessment[3]	B	0.30 (0.14)			
	B1	0.35 (0.15)	0.05 (0.02, 0.07)	.002	0.69
	B2	0.28 (0.16)	−0.03 (−0.05, 0.00)	.077	−0.39
	B3	0.30 (0.16)	−0.01 (−0.04, 0.02)	.622	−0.11
	B4	0.28 (0.16)	−0.02 (−0.05, 0.01)	.132	−0.44
	B5	0.31 (0.16)	0.01 (−0.02, 0.04)	.530	0.15
	R1	0.35 (0.14)	0.05 (0.02, 0.07)	.001	0.37
	R2	0.36 (0.15)	0.05 (0.03, 0.08)	<.001	0.36
	R3	0.37 (0.16)	0.06 (0.04, 0.09)	<.001	0.40
	R4	0.30 (0.13)	0.00 (−0.03, 0.03)	.963	0.00
	R5	0.34 (0.14)	0.03 (0.01, 0.06)	.019	0.25

[1] Age and medication included as covariates in the linear mixed model

[2] Cohens d

[3] Segments for baseline (B1–B5) and risk scenario (R1–R5) with the mean of baseline (B) as the reference category for this analysis

BCPD: brightness corrected pupil diameter

Supplementary Table 3. Results of linear mixed model analysis of main effect of VR–CE on HRV parameters.

		**HRV**[1]			
			Linear mixed effects model analysis		
HRV parameter	Assessment[2]	Mean (SD)	β (95% CI)	*p*–value	Effect size[3]
**HFB**	B	377.48 (3453.34)			
	R	59-03 (96.95)	–318.45 (–926.94, 290.03)	.303	–0.09
**VLFB**	B	48.51 (48.76)			
	R	57.22 (65.96)	8.71 (–2.54, 19.96)	.128	0.14
**LFB**	B	80.90 (154.95)			
	R	78.63 (86.41)	–2.26 (–30.13, 25.60)	.873	–0.01
**VHFB**	B	1512.69 (16353.90)			
	R	12.25 (29.69)	–1500.44 (–4377.56, 1376.68)	.305	–0.09
**LF/(LF+HF)**	B	0.61 (0.19)			
	R	0.64 (0.18)	0.03 (–0.00, 0.06)	.076	0.19
**HF/(LF+HF)**	B	0.39 (0.19)			
	R	0.36 (0.18)	–0.03 (–0.06, 0.00)	.076	–0.19
**LF/HF**	B	2.64 (3.13)			
	R	2.79 (2.86)	0.15 (–0.44, 0.74)	.613	0.05
**Total power**	B	267381.75 (2570613.70)			
	R	26964.12 (27946.60)	–240417.63 (–692363.44, 211528.18)	.295	–0.09
**SDRR**	B	43.99(44.29)			
	R	41.59 (22.69)	–2.39 (–10.04, 5.25)	.538	–0.06
**RMSSD**	B	31.01 (28.93)			
	R	31.24 (25.34)	0.23 (–4.74, 5.20)	.928	0.01
**SDSD**	B	31.01 (28.93)			
	R	31.24 (25.34)	0.23 (–4.74, 5.20)	.928	0.01
**pNN50**	B	6.39 (9.84)			
	R	6.56 (9.33)	0.16 (–0.88, 1.20)	.758	0.04
**SD1**	B	0.04 (0.16)			
	R	0.02 (0.02)	–0.02 (–0.04, 0.01)	.314	–0.09
**SD2**	B	0.05 (0.03)			
	R	0.05 (0.03)	0.00 (–0.00, 0.01)	.172	0.16
**SD1/SD2**	B	0.43 (0.27)			
	R	0.42 (0.24)	–0.01 (–0.05, 0.04)	.772	–0.03

[1] Gender, age, ambient temperature, medication, cci-score and smoking included as covariates in the linear mixed model

[2] Baseline (B) and risk scenario (R) with baseline as the reference category for this analysis

[3] Cohens d

HRV: heart rate variability, HFB: high frequency band, VLFB: very low frequency band, LFB: low frequency band, VHFB: very high frequency band, LF/(LF+HF): ratio between low frequency and total between low frequency and high frequency, HF/(LF+HF): ratio between high frequency and total between low frequency and high frequency, LF/HF : ratio between low frequency and high frequency, SDRR: standard deviation of R-R intervals, RMSSD: root mean square of successive R-R differences, SDSD: standard deviation of successive differences in R-R intervals, pNN50: percentage of successive RR intervals differing by more than 50ms, SD1: standard deviation of the distance of each point from the y=x axis, SD2: standard deviation of each point from the y=x + average R-R Interval, SD1/SD2: SD1/SD2 ratio

Supplementary Table 4. Correlation matrix of subjective and physiological craving.

	1VAS	3VAS	5VAS	1SCL	2SCL	3SCL	4SCL	5SCL	1 NS.SCR	2 NS.SCR	3 NS.SCR	4 NS.SCR	5 NS.SCR	1HR	2HR	3HR	4HR	5HR	1RR	2RR	3RR	4RR	5RR	1BCPD	2BCPD	3BCPD	4BCPD	5BCPD
**1VAS**																												
**3VAS**	0.59***																											
**5VAS**	0.49***	0.82***																										
**1SCL**	0.14	0.23*	0.15																									
**2SCL**				0.76***																								
**3SCL**	0.03	0.20*	0.08	0.67***	0.91***																							
**4SCL**				0.53***	0.74***	0.86***																						
**5SCL**	0.08	0.12	0.01	0.53***	0.69***	0.77***	0.85***																					
**1NS.SCR**	0.08	0.24*	0.20*	0.39***	0.56***	0.52***	0.40***	0.40***																				
**2NS.SCR**				0.16	0.32***	0.40***	0.42***	0.38***	0.49***																			
**3NS.SCR**	0	0.21*	0.21*	0.14	0.20*	0.27**	0.32***	0.34***	0.35***	0.37***																		
**4NS.SCR**				0.07	0.13	0.20*	0.34***	0.39***	0.21*	0.39***	0.36***																	
**5NS.SCR**	-0.02	-0.01	0.09	-0.01	0.1	0.15	0.18	0.24*	0.26**	0.19*	0.26**	0.39***																
**1HR**	0.03	0.20*	-0.01	0.26**	0.43***	0.36***	0.32***	0.30**	0.35***	0.09	0.14	0.17	0.18															
**2HR**				0.18	0.29**	0.29**	0.27**	0.29**	0.08	0.17	0.09	0.15	-0.09	0.54***														
**3HR**	0.11	0.14	0.03	0.11	0.14	0.21*	0.17	0.16	0.13	0.1	0.20*	0.14	0.03	0.41***	0.58***													
**4HR**				0.09	0.14	0.17	0.18	0.24*	0.16	0.12	0.08	0.26**	0.18	0.39***	0.48***	0.57***												
**5HR**	0.1	0.12	0.03	-0.05	-0.05	-0.06	-0.08	-0.02	0.03	-0.08	-0.02	0.06	0.20*	0.14	0.13	0.31***	0.43***											
**1RR**	-0.02	0.01	0.02	-0.14	-0.11	-0.03	0.07	-0.05	0.01	0	0.02	-0.02	-0.09	0.04	0.06	0.07	0.13	0.07										
**2RR**				-0.14	-0.11	-0.05	-0.01	-0.11	-0.16	-0.19*	0	-0.1	-0.07	0.14	0.1	0.12	0.23*	0.14	0.33***									
**3RR**	-0.05	-0.01	0.01	0.05	0.04	0.1	0.15	0.12	-0.18	-0.11	-0.01	0.09	-0.15	0.22*	0.34***	0.20*	0.26**	0.05	0.26**	0.37***								
**4RR**				-0.08	-0.11	-0.08	-0.08	-0.12	-0.19*	-0.03	-0.02	-0.07	-0.20*	0.07	0.1	0.18	0.21*	0.05	0.23*	0.37***	0.46***							
**5RR**	0.03	0.12	0.03	-0.07	-0.12	-0.07	-0.02	-0.04	-0.09	-0.1	0.16	0.01	-0.18	0	0.15	0.16	0.11	0.14	0.30**	0.33***	0.34***	0.27**						
**1BCPD**	-0.02	-0.06	-0.11	0.01	-0.09	-0.05	0.04	0.03	-0.06	-0.12	0.11	0.01	-0.05	0.09	0.07	0.07	-0.03	-0.03	-0.01	0.24**	0	0.01	0.15					
**2BCPD**				0.09	-0.02	0.01	0.05	0	-0.02	-0.08	0.15	0.02	0.02	0.09	-0.01	-0.02	-0.03	0.05	0.13	0.1	-0.02	0.08	0.19*	0.64***				
**3BCPD**	-0.08	-0.01	0.14	0.04	-0.08	-0.06	-0.02	-0.04	-0.06	0.02	0.20*	0.03	0	0.05	0.20*	0.12	-0.02	0.13	0.08	0.04	0.01	0.12	0.1	0.42***	0.63***			
**4BCPD**				-0.05	-0.02	0.06	0.09	0.02	0.02	0.05	0.12	0	0.14	0.18	0.11	0.1	0.09	0.09	0.15	0.11	0	0.09	0.11	0.49***	0.62***	0.65***		
**5BCPD**	-0.18	0.01	0.12	-0.01	0.01	0.04	-0.01	-0.08	-0.01	0.05	0.11	-0.03	0.05	0.16	0.15	0.1	-0.03	0.04	0.06	0.04	-0.02	0.09	0.08	0.40***	0.50***	0.69***	0.66***	

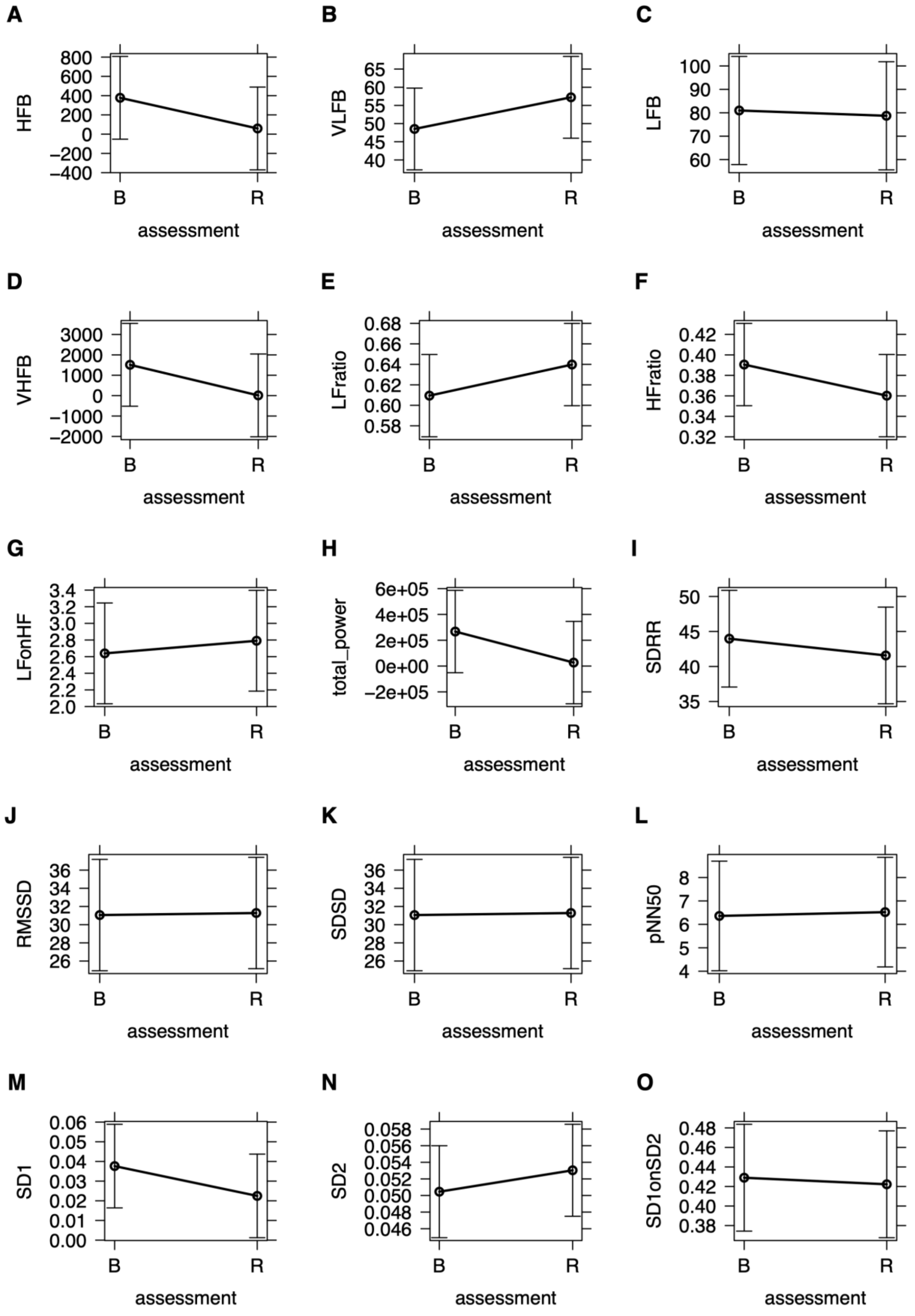

Supplementary Figure 1. Linear mixed-effect models displaying main effects of VR-CE over time on different HRV parameters. Plots of estimated marginal means and 95% confidence intervals of the means obtained from linear mixed-effect models displaying nonsignificant main effects by VR-CE over time on HFB in ms^2 (A), VLFB in ms^2 (B), LFB in ms^2 (C), VHFB in ms^2 (D), LF/(LF+HF) (E), HF/(LF+HF) (F), LF/HF (G), total power in

ms^2 (H), SDRR in ms (I), RMSSD in ms (J), SDSD in ms (K), pNN50 in % (L), SD1 (M), SD2 (N) and SD1/SD2 (O). Depicted are baseline (B) and risk scenario (R) with baseline (B) as the reference category for this analysis.
HFB: high frequency band, VLFB: very low frequency band, LFB: low frequency band, VHFB: very high frequency band, LF/(LF+HF): ratio between low frequency and total between low frequency and high frequency, HF/(LF+HF): ratio between high frequency and total between low frequency and high frequency, LF/HF : ratio between low frequency and high frequency, SDRR: standard deviation of R-R intervals, RMSSD: root mean square of successive R-R differences, SDSD: standard deviation of successive differences in R-R intervals, pNN50: percentage of successive RR intervals differing by more than 50ms, SD1: standard deviation of the distance of each point from the y=x axis, SD2: standard deviation of each point from the y=x + average R-R Interval, SD1/SD2: SD1/SD2 ratio
